# Cardiac injury and mortality in patients with Coronavirus disease 2019 (COVID-19): insights from a mediation analysis

**DOI:** 10.1007/s11739-020-02495-w

**Published:** 2020-09-27

**Authors:** Alberto Cipriani, Federico Capone, Filippo Donato, Leonardo Molinari, Davide Ceccato, Alois Saller, Lorenzo Previato, Raffaele Pesavento, Cristiano Sarais, Paola Fioretto, Sabino Iliceto, Dario Gregori, Angelo Avogaro, Roberto Vettor

**Affiliations:** 1 Department of Cardio-Thoraco-Vascular Sciences and Public Health, University of Padova, Via Giustiniani, 2, 35128 Padua, Italy; 2 Department of Medicine, University of Padova, Via Giustiniani, 2, Padua, 35128 Italy; 3 Azienda Ospedaliera, University of Padua, Via Giustiniani, 2, Padua, 35128 Italy

**Keywords:** Coronavirus 2019, COVID-19, Cardiac injury, Cardiac troponin

## Abstract

**Backgrounds:**

Patients at greatest risk of severe clinical conditions from coronavirus disease 2019 (COVID-19) and death are elderly and comorbid patients. Increased levels of cardiac troponins identify patients with poor outcome. The present study aimed to describe the clinical characteristics and outcomes of a cohort of Italian inpatients, admitted to a medical COVID-19 Unit, and to investigate the relative role of cardiac injury on in-hospital mortality.

**Methods and results:**

We analyzed all consecutive patients with laboratory-confirmed COVID-19 referred to our dedicated medical Unit between February 26th and March 31st 2020. Patients’ clinical data including comorbidities, laboratory values, and outcomes were collected. Predictors of in-hospital mortality were investigated. A mediation analysis was performed to identify the potential mediators in the relationship between cardiac injury and mortality. A total of 109 COVID-19 inpatients (female 36%, median age 71 years) were included. During in-hospital stay, 20 patients (18%) died and, compared with survivors, these patients were older, had more comorbidities defined by Charlson comorbidity index ≥ 3(65% vs 24%, *p* = 0.001), and higher levels of high-sensitivity cardiac troponin I (Hs-cTnI), both at first evaluation and peak levels. A dose–response curve between Hs-cTnI and in-hospital mortality risk up to 200 ng/L was detected. Hs-cTnI, chronic kidney disease, and chronic coronary artery disease mediated most of the risk of in-hospital death, with Hs-cTnI mediating 25% of such effect. Smaller effects were observed for age, lactic dehydrogenase, and d-dimer.

**Conclusions:**

In this cohort of elderly and comorbid COVID-19 patients, elevated Hs-cTnI levels were the most important and independent mediators of in-hospital mortality.

**Electronic supplementary material:**

The online version of this article (10.1007/s11739-020-02495-w) contains supplementary material, which is available to authorized users.

## Introduction

The severe acute respiratory syndrome coronavirus 2 (SARS-CoV-2) infection shows fast contagiousness and a high rate of morbidity and mortality [1–4]. Italian public healthcare systems have been overwhelmed by infected people, forcing clinicians to tough decisions for rationing medical care [5, 6]. Assessing the severity of coronavirus disease 2019 (COVID-19) is crucial for a correct triage and prioritization. From published experiences, the patients at greatest risk of serious illness of COVID-19 and death are elderly comorbid patients, particularly those with cardiovascular diseases [7–10]. Cardiac injury, demonstrated by the rise of high-sensitivity cardiac troponins is a common finding in patients with severe COVID-19 and previous reports pointed it as a strong predictor of adverse outcome [9–12]. Such observations may justify considering other pathogenetic mechanisms of myocardial damage, beyond the direct viral infection of the myocardium by SARS-CoV-2. Either the concomitant presence of the cytokines storm and the pneumonia-related hypoxia could also determine myocardial ischemia, by altering the myocardial oxygen supply–demand balance. The concerted negative effects of these events, along with underlying coronary artery disease (CAD), can explain the cardiac troponins elevation. However, little is known about the clinical significance of cardiac injury in non-critically ill COVID-19 patients. Furthermore, the link between cardiac injury and mortality is unclear: specifically, it is not known whether there is a direct effect of cardiac injury on mortality or whether this effect is mediated by the underlying comorbid conditions.

Therefore, the aims of the present study are (1) to describe the clinical characteristics, the incidence of cardiac injury and outcomes of a cohort of Italian COVID-19 patients, admitted to our medical non-intensive COVID-19 Unit, (2) to perform a post-hoc exploratory mediation analysis to identify the extent to which the cardiac injury mediates in-hospital mortality.

## Methods

The data, analytic methods, and study materials will be available to other researchers for purposes of reproducing the results or replicating the procedure on reasonable request.

This was a single-center, observational study enrolled all consecutive patients with laboratory-confirmed SARS-CoV-2 infection and clinical and radiological signs of COVID-19, referred from the Emergency Department to our medical, non-intensive COVID-19 Unit of Azienda Ospedaliera Padova (Italy), between February 26th and March 31st 2020. Criteria for referral to our Unit was the no-need, or no-eligibility for age or comorbidities, of mechanical or non-invasive ventilation. Patients without laboratory cardiac troponins evaluation were excluded from the study.

According to the WHO guidance (13), laboratory confirmation for SARS-CoV-2 was defined as a positive result of real-time reverse transcriptase-polymerase chain reaction assay of nasal and pharyngeal swabs.

Study data and clinical information were collected and managed by medical staff using REDCap electronic data capture tools hosted at University of Padova [14, 15].

All clinical investigations were conducted according to the principles expressed in the Declaration of Helsinki (2001). Verbal consent from patients for management of personal data was obtained. The study protocol was approved by the cardiovascular section inhouse Ethics Committee on Human Research of the Padova Province.

### Data collection

Clinical patient data reported in this study included the following: age, sex, body mass index (BMI), clinical parameters on admission in the Emergency Room, comorbidities including hypertension, atrial fibrillation, diabetes mellitus, dyslipidemia, chronic CAD, chronic heart failure, cerebrovascular disease, peripheral atheromasia, chronic pulmonary- kidney-liver disease, solid malignancy, leukemia, lymphoma, dementia, connective tissue disease and acquired immunodeficiency disease. Charlson Comorbidity Index, a score widely used in epidemiological studies to predict length of hospitalization, health-resources use, and 1-year mortality, was calculated [16]. Laboratory examinations on admission and the maximum value during the hospitalization were reported. In particular, the high-sensitivity cardiac troponin I (Hs-cTnI) assay was based on the immunometric method with chemiluminescence detection and patients were considered to have acute cardiac injury if serum levels Hs-cTnI were above the 99th percentile upper reference limit (32 ng/L for males, 16 ng/L for females). Electrocardiography and echocardiography data were not object of analysis in this work. Cardiac imaging diagnostic techniques requiring the transportation of COVID-19 patients in other areas of the hospital and all cardiac catheterization laboratory procedures were discouraged to minimize the risk of dissemination and staff exposure, and performed only if considered decisive for the patient management or lifesaving, according to scientific consensus statements [17, 18].

### Outcome

The endpoint was incidence of COVID-19 associated in-hospital mortality. The number of patients discharged, and referred to intensive care unit (ICU), and still admitted on April the 1st 2020, was also determined. Discharge criteria were: (1) relieved clinical symptoms, (2) normal body temperature, (3) complete weaning from oxygen therapy maintained for 48 h, (4) resolution of inflammation as shown by chest radiography and blood tests, (5) no evidence of arterial oxygen desaturation on six-minute walk test.

### Statistical analysis

Descriptive statistics were carried out for all clinical variables. Data are presented as median (interquartile range [IQR]) for continuous variables and as percentages (absolute numbers) for categorical variables. To compare differences between survivors and non-survivors, we used the Mann–Whitney *U* test, Chi-square test, or Fisher’s exact test, as appropriate.

A two-sided *p* of less than 0.05 was considered statistically significant.

Cardiac injury effect on in-hospital mortality was evaluated using a Generalized Additive Model with logit link and restricted cubic splines to allow for non-linearities [19]. Potential mediators of the link between cardiac injury and mortality were explored using a mediation analysis [20]. Both mediation and outcome models were built using an exhaustive search approach among all variables considered in the dataset using the Bayesian Information Criterion [21]. All confidence intervals (CIs) have been computed using bias-adjusted bootstrap with 10,000 runs. We used the Somer’s Dxy as a measure of goodness of fit (the closer to 1 the better) to present results of the multivariable model for outcome. Analyses were conducted using the R System [22], with mediation [23] and glmulti [24] libraries.

## Results

### Baseline characteristics

During the study period, 136 patients were admitted to our Medical Unit for COVID-19. Patients with missing Hs-cTnI results (*n* = 27) were excluded. The study population included 109 patients (female *n* = 36, 33%), with a median age was 71 years (IQR 60–81), and a BMI 28 kg/m^2^ [24–30]. During the observation period, 20 (18%) patients died, 83 (76%) patients were discharged, 6 (5.5%) were still hospitalized. The median length of stay was 11 days (IQR 6–16).

Hypertension (68 patients, 62%), dyslipidemia (39, 36%) and diabetes mellitus (27, 25%) were the most common cardiovascular risk factors. No current smokers were identified, past smokers were 26 (24%). Chronic CAD, chronic heart failure, cerebrovascular disease, and chronic kidney disease were known in 18 patients (17%), 16 (15%), 17 (16%), and 13 (12%), respectively. Charlson Comorbidity Index was 0 in 36 (33%), 1–2 in 39 (36%), > 3 in 34 (16%). Other comorbidities, as well as admission parameters, symptoms, home therapy and clinical management are reported in Supplemental Files.

Compared with the survivors, non-survivors were significantly older (86 vs 69 years, *p* < 0.001), more likely to have hypertension (80% vs 58%,* p* = 0.050), dyslipidemia (55% vs 32%, *p* = 0.047), chronic kidney disease (30% vs 8%, *p* = 0.014), chronic CAD (45% vs 9%, *p* = 0.001) and higher Charlson Comorbidity Index values (Table 1).

**Table 1:** Demographic and clinical characteristics of patients with COVID-19

	COVID-19	Alive	Dead	*p*
	*n* = 109	*n* = 89	*n* = 20	
Age (years)	71 (60–81)	69 (57–79)	86 (77–87)	< 0.001
Female sex	36 (33)	26 (29)	10 (50)	0.074
BMI (kg/m^2^)	28 (24–31)	28 (25–31)	22 (19–31)	0.071
**Comorbidities**				
Hypertension	68 (62)	52 (58)	16 (80)	0.050
Diabetes mellitus	27 (25)	21 (24)	6 (30)	0.572
Complicated diabetes	16 (15)	5 (8)	11 (24)	0.020
Dyslipidaemia	39 (36)	28 (32)	11(55)	0.047
Smoking history	26 (24)	20 (23)	6 (30)	0.475
Chronic kidney disease	13 (12)	7 (8)	6 (30)	0.014
Chronic liver disease	7 (6)	7 (8)	0	0.345
Cerebrovascular disease	17 (16)	12 (14)	5 (25)	0.302
Chronic heart failure	16 (15)	11(12)	5 (25)	0.167
Chronic CAD	18 (17)	9 (10)	9 (45)	0.001
Charlson Comorbidity Index ≥ 3	34 (31)	21 (24)	13 (65)	0.001
**Admission laboratory findings**				
Hs-cTnI (ng/L)	17.0 (5.0–54.0)	6.0 (3.0–14.0)	64.0 (36.0–133)	< 0.001
Abnormal Hs-cTnI	41 (38)	23 (26)	18 (90)	< 0.001
Hemoglobin (g/dL)	128 (122–140)	129 (123–143)	124 (116–131)	0.111
Lymphocytes (× 10^9^/L)	0.97 (0.70–1.33)	1.07 (0.73–1.38)	0.66 (0.40–1.05)	0.079
CRP (mg/L)	73 (37–120)	58 (30–118)	101 (80–161)	0.006
d-Dimer (µg/L)	275 (158–784)	230 (150–575)	773 (336–1711)	0.003
LDH (U/L)	326 (249–406)	304 (245–381)	405 (294–670)	0.008
**Peak laboratory findings**				
Hs-cTnI (ng/L)	18.0 (7.0–96.0)	16.0 (6.0–37.0)	279 (48–1084)	< 0.001
Abnormal Hs-cTnI	46 (42)	28 (32)	18 (90)	< 0.001
Hemoglobin (g/dL)	11.1 (9.60–12.6)	11.2 (9.8–12.6)	10.9 (8.1–12.5)	0.320
Lymphocytes (× 10^9^/L)	0.70 (0.50–1.00)	0.80 (0.50–1.10)	0.45 (0.20–0.75)	0.002
CRP (mg/L)	130 (87–200)	120 (74–170)	185 (110–251)	0.045
d-Dimer (µg/L)	901 (361–2883)	580 (293–2106)	2846 (1092–4029)	0.003
LDH (U/L)	397 (317–580)	366 (294–517)	630 (484–1098)	< 0.001
Serum ferritin (µg/L)	1225 (667–2311)	1070 (665–1978)	2663 (756–4701)	0.035
BNP (ng/L)	90 (22–262)	70 (20–190)	210 (98–351)	0.034
IL-6 (ng/L)	48 (15–115)	40 (11.8–95)	164 (56–298)	0.028
**Outcome**				
Length of stay (days)	11 (6–16)	12 (6–17)	8 (4–14)	0.129
Referred to ICU	31 (30)			
Death	20 (18)			
Still hospitalized	6 (5.5)			
Discharged	83 (76)			

Categorical variables are presented as number of patients (%). Continuous values are expressed as median with 25% and 75%-iles

Abnormal Hs-cTnI was defined when ≥ 32 ng/L for males, ≥ 16 ng/L for females

*BMI* body mass index, *CAD* coronary artery disease, *Hs-cTnI* high-sensitivity cardiac troponin I, *CRP* C-reactive protein, *LDH* lactate dehydrogenase, *BNP* brain natriuretic peptide, *IL-6* interleukin-6, *ICU* intensive care unit

### Laboratory value

**Fig. 1:**
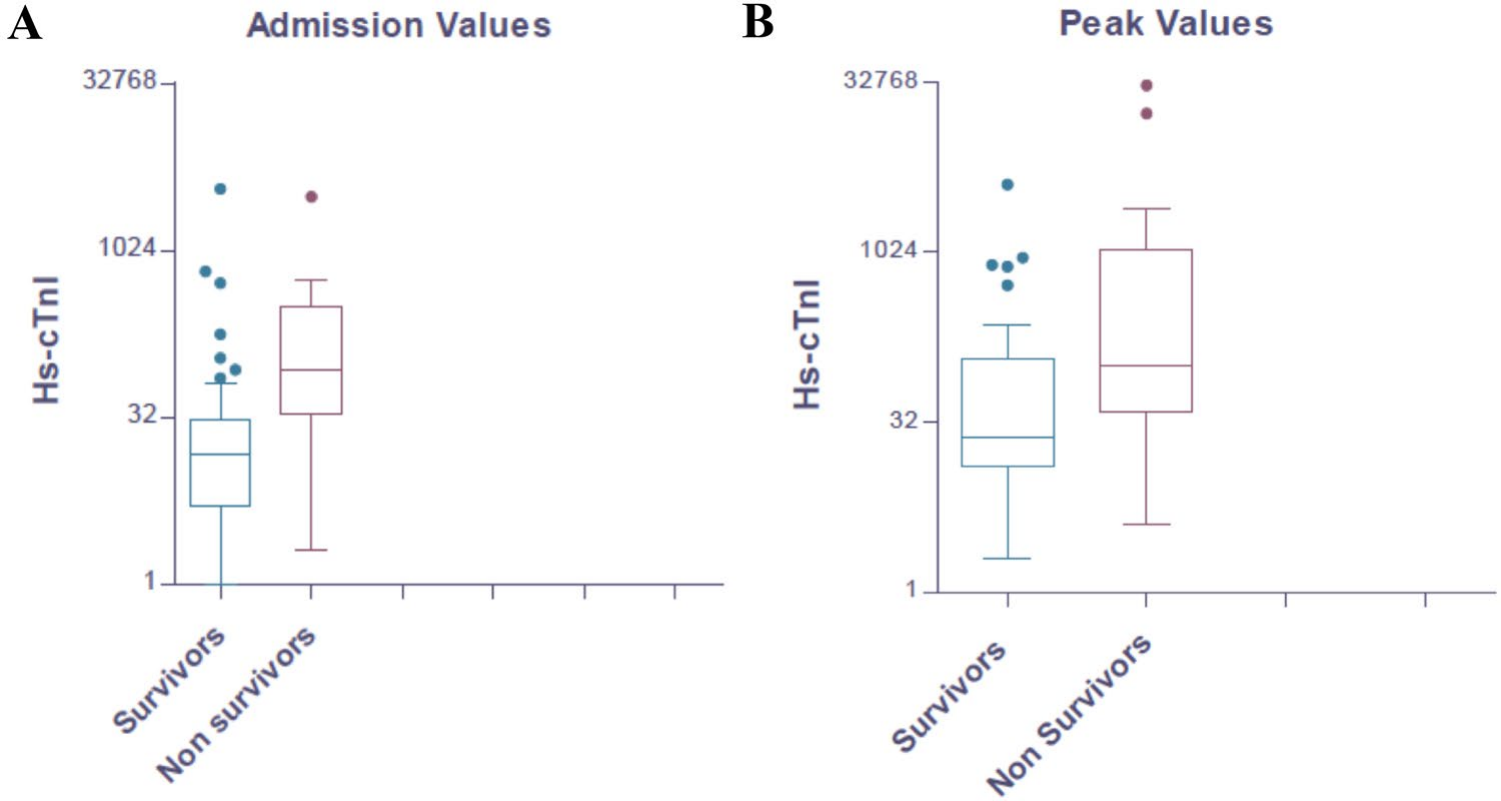
Levels of admission and peak Hs-cTnI in our cohort of COVID-19 patients. Compared with survivors, non-survivors presented higher median levels of Hs-cTnI both on admission (64 vs 6 ng/L, *p* < 0.001) (**a**) and peak levels during the hospitalization (279 vs 16 ng/L, *p* < 0.001) (**b**)

On admission, abnormal median values of hemoglobin, lymphocytes, C-reactive protein (CRP), d-Dimer and lactate dehydrogenase (LDH) were recorded. Hs-cTnI levels were out of normality ranges in 41 (38%). Compared with survivors, non-survivors presented with higher median levels of Hs-cTnI (64 vs 6 ng/L, *p* < 0.001; abnormal in 90% vs 30%, *p* < 0.001) (Fig. 1a), CRP (101 vs 58 mg/L, *p* = 0.006), d-Dimer (773 vs 230 µg/L, *p* = 0.003) and LDH (405 vs 304 U/L, *p* = 0.008). During the hospitalization, almost the same trend was observed, with non-survivors showing higher levels of peak Hs-cTnI (279 vs 16 ng/L, *p* < 0.001; abnormal in 90% vs 32%, *p* < 0.001) (Fig. 1b), lymphocytes (0.45 vs 0.80 × 10^9^/L, *p* = 0.002), CRP (185 vs 20 mg/L, *p* = 0.045), d-Dimer (2846 vs 580 µg/L, *p* = 0.003), LDH (630 vs 366 U/L, *p* < 0.001). Also, serum ferritin (2663 vs 1070 µg/L, *p* = 0.035), brain natriuretic peptide (BNP) (210 vs 70 ng/L, *p* = 0.034) and interleukin-6 (IL-6) (164 vs 40 ng/L, *p* = 0.028) were higher in non-survivors, compared with survivors.

### Cardiac injury effect on in-hospital mortality

**Fig. 2:**
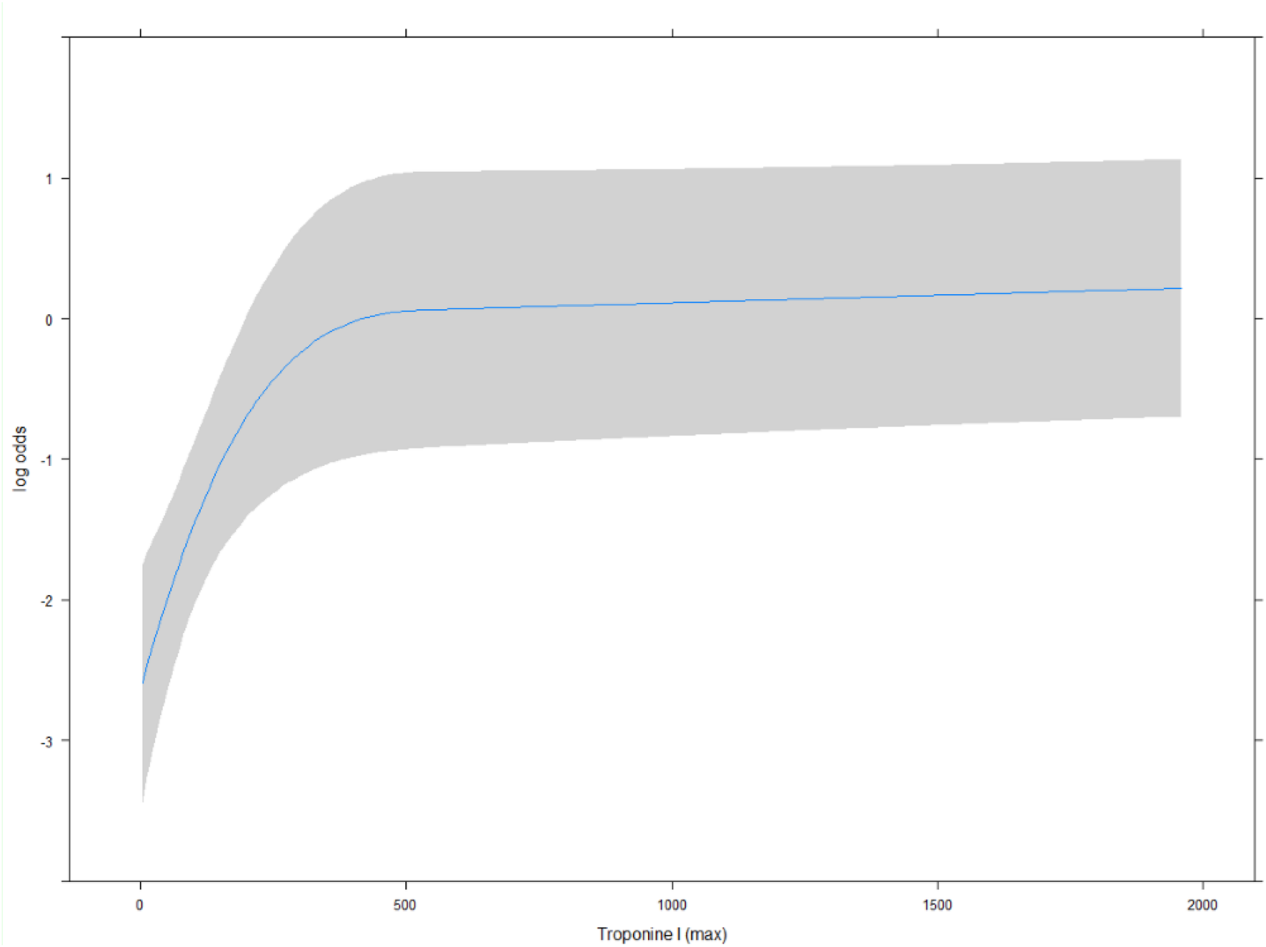
Effect of peak Hs-cTnI on mortality. Non-linear effect (*p* < 0.001) of Hs-cTnI from 0 to 200 ng/L is to increase risk of death by odds ratio (OR) 6.92 (95% CI 2.39–19.99). Curvature changes to a plateau at 209.18 ng/L (95% CI 110.80–365.75)

Figure 2 indicates the effect of peak Hs-cTnI on mortality: a non-linear effect (*p* < 0.001) of Hs-cTnI levels on in-hospital mortality was observed from 0 to 200 ng/L [OR 6.92 (95% CI 2.39–19.99)].

**Fig. 3:**
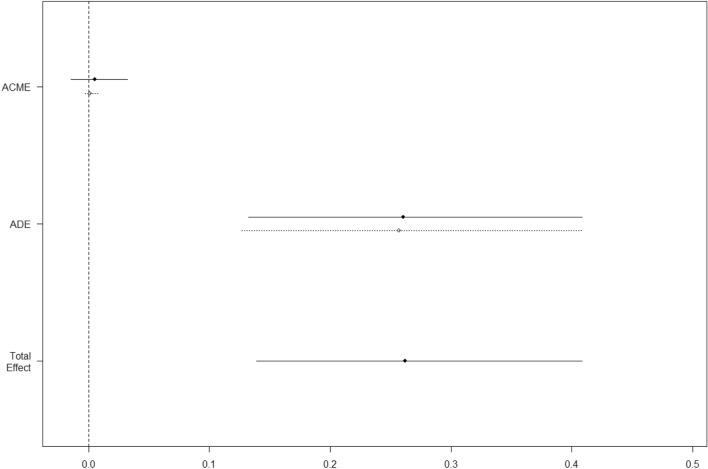
Mediation analysis of an abnormal Hs-cTnI. Average causal mediation effect (ACME) and average direct effect (ADE). Effects are the increase (decrease) in probability of the outcome between risk factor’s levels

Table 2 reports the relative effect of each predictor as a mediator on in-hospital mortality as evaluated at multivariate mediation analysis: as it can be seen the strongest mediator of in-hospital mortality was peak Hs-cTnI, followed by chronic kidney disease, and chronic CAD. These three factors led to a proportion mediated of 70%. The addition of LDH increased the proportion mediated to 89%. The independent predictive effect of Hs-cTnI is further supported by the multivariate model for mortality with an OR of 32.58 (Table 3). We then performed an additional analysis to assess whether the peak of Hs-cTnI was either mediated or direct. Figure 3 shows that a statistically significant effect of the average direct effect: this testifies a direct effect of peak Hs-cTnI on mortality, with no significant interaction with other confounders.

**Table 2:** Mediation analysis of risk of death in patients with COVID-19

	Effect	95%	CI	*p* value	ME (%)
Abnormal Hs-cTnI	0.25	0.12	0.39	0.004	0.57
Female sex	0.09	− 0.04	0.23	0.13	6.98
Age > 70 years	0.14	0.00	0.27	0.048	0.51
Hypertension	0.04	− 0.09	0.17	0.462	3.21
Diabetes	0.06	− 0.08	0.22	0.376	4.67
Dyslipidemia	0.08	− 0.05	0.23	0.224	3.72
Chronic kidney disease	0.24	0.01	0.52	0.044	0.00
Chronic CAD	0.21	0.02	0.45	0.022	0.60
Heart failure	0.00	− 0.13	0.19	0.88	18.17
Charlson Comorbidity Index ≥ 3	0.09	− 0.06	0.28	0.25	4.52
P/F < 100	− 0.11	− 0.27	0.02	0.108	5.32
LDH	0.19	0.05	0.33	0.012	1.21
d-Dimer	0.14	− 0.01	0.30	0.058	2.64
Serum ferritin	− 0.06	− 0.18	0.08	0.374	0.57
BNP	0.08	− 0.07	0.21	0.216	5.87
Lymphocytes	− 0.14	− 0.30	0.00	0.048	1.96
Hemoglobin	− 0.03	− 0.16	0.10	0.684	3.30

Direct effects of each predictor on mortality as evaluated at mediation analysis. No mediated effect was observed as statistically significant. Effect is the increase (decrease) in probability of the outcome between risk factor’s levels. ME is the size (%) of the average causal mediation effects relative to the total effect

*Hs-cTnI* high-sensitivity cardiac troponin I, *CAD* coronary artery disease, *LDH* lactate dehydrogenase, *BNP* brain natriuretic peptide

**Table 3:** Multivariable model for mortality

	OR	95% CI	*p* value
LDH (270 U/L increase)	2.70	1.20–6.08	0.017
Abnormal Hs-cTnI	32.58	3.50–303.31	0.030
Chronic coronary artery disease	5.20	1.18–22.94	0.008

Somer’s Dxy 0.78

Multivariate model showing the independent predictive effect of cardiac injury

*LDH* lactate dehydrogenase, *Hs-cTnI* high-sensitivity cardiac troponin I

## Discussion

Cardiac injury is a powerful predictor of mortality in patients with severe COVID-19 infection. However, little is known in non-critically ill COVID-19 patients. Furthermore, it is unclear the relative predictive role of cardiac injury in the context of other comorbidities.

In the present study, we show that: (1) COVID-19 non-survivors show higher Hs-cTnI, both on admission and peak levels. (2) There is a dose–response relationship between Hs-cTnI levels and mortality up to 200 ng/L; (3) Hs-cTnI is the most powerful mediator of in-hospital mortality; (4) This mediation is independent of other underlying confounding variables.

Since the first Chinese observational studies, cardiac injury demonstrated by elevation of cardiac troponins has been reported in 10–30% in-patients with COVID-19 and associated with severe COVID-19 illness, a higher need for intensive care and higher odds of in-hospital mortality [3, 4, 8, 9]. Our data confirmed this observation, yet reporting a higher rate of cardiac injury (38% on admission, 46% during hospitalization) and mortality (18%), which may be explained by the characteristics of our population, represented by older and comorbid patients. Indeed, median age of our patients (71 years) was notably higher than Huang et al. (49 years), Zhou et al. (56 years), Guo et al. (58.5 years), and Shi et al. (64 years) [3, 4, 8, 9]. Along with the age, also comorbidities were more prevalent, with a Charlson comorbidity index ≥ 3 in about one third of patients, and in keeping with previous reports [4, 8, 25, 26], patients with hypertension, diabetes, chronic kidney disease and chronic CAD were associated with poor outcome.

Cardiac injury in patients with COVID-19 has been also demonstrated to be predictive of in-hospital mortality [7, 12, 27, 28]. In a metanalysis exploring the risk factors for COVID-19 patients to develop critical disease or death, a Hs-cTnI > 28 pg/mL was associated with an OR of 43.24 (95% CI 9.92–188.49) to deterioration of the patient’s condition [27]. We also found that the risk for mortality increases exponentially with low levels of Hs-cTnI, reaching a plateau at a concentration of about 200 ng/L. These Hs-cTnI levels are uncommonly observed in type-1 acute myocardial infarction or acute myocarditis, and seem likely to be related to myocardial inflammation and supply/demand discrepancy (ischemia).

In the context of elderly and comorbid patients affected by viral pneumonia and systemic inflammation, it can however be problematic to extrapolate the clinical predictive value of cardiac injury, beyond other factors like hypoxemia, hypercoagulation, and systemic inflammation. This is clinically important since experts’ consensus endorse the use of Hs-cTnI after hospitalization as a tool to identify COVID-19 patients with cardiac injury, predict the progression of the disease and set up early treatment approach [29, 30]. Our mediation analysis highlights the central role of cardiac injury in the prediction of mortality, since the peak of Hs-cTnI was the variable with the largest impact on the odds for in-hospital mortality.

The present study has the limitations inherent of single-center, retrospective, observational studies, with a relatively small sample size. Nevertheless, the available sample allows to estimate, assuming a baseline event rate of 20%, a range of OR between approximately 0.3 and 1.8 with a power of 0.80 and the observed effects are all well above the upper limit. We included all patients admitted to our medical COVID-19 Unit for a COVID illness, who did not need any positive-pressure ventilation or were deemed ineligible to it for age or multiple comorbidities; so, our data may not extend to the entire patients with COVID-19, in particular, the most critical patients admitted to ICU. Autopsy cardiovascular studies are needed to advance our knowledge and understanding the pathophysiology of cardiac injury in COVID-19.

In conclusion, the risk stratification is of crucial importance in patients suffering from COVID-19: the use of Hs-cTnI is now considered an important tool to predict outcome. In this study, we not only confirmed that Hs-cTnI is a predictor of in-hospital mortality also in non-critically ill patients with COVID-19, but also that it is the most important mediator independent from any other confounder. Cardiac damage plays a central role in the prognosis of patients with COVID-19 infection.

## Electronic supplementary material

Below is the link to the electronic supplementary material.

Supplementary file1 (DOCX 16 kb)

## Data Availability

The data, analytical methods, and study materials will be available to other researchers for purposes of reproducing the results or replicating the procedure on reasonable request.
