# Supplementary material for: Cardiac injury and mortality in patients with Coronavirus disease 2019 (COVID-19): insights from a mediation analysis
Source: Intern Emerg Med. 2020 Sep 27;16(2):419–27. doi: 10.1007/s11739-020-02495-w (PMC7520162; doi:10.1007/s11739-020-02495-w)
Supplement: Supplementary file 1 — Supplementary file1 (DOCX 16 kb) [file 11739_2020_2495_MOESM1_ESM.docx]

**SUPPLEMENTAL FILES**

**Supplemental Results**

**OTHER COMORBIDITIES.** Patients with atrial fibrillation were 21 (19%), peripheral atheromasia in 23 (21%), chronic pulmonary disease 17 (16%), chronic kidney disease 13 (12%), chronic liver disease 7 (6%), solid malignancy 16 (15%), leukemia 1 (0.9%), lymphoma 1 (0.9%), dementia 17 (16%), connective tissue disease 6 (6%) and acquired immunodeficiency disease 0 (0%). Compared with the survivors, non-survivors had more frequently peripheral atheromasia (40% vs 17%, p=0.033) (Table 1).

**HOME THERAPY**. With regard to home therapy, 47 patients (43%) were taking ACEi or ARB, 25 (23%) betablockers, 28 (26%) statins, 21 (19%) diuretics, 25 (23%), cardioaspirin, 10 (9%) P2Y12 inhibitors, 13 (12%) oral anticoagulants. Compared with the survivors, non-survivors were more frequently on betablockers (45% vs 18%, p=0.011) and diuretics (50% vs 12%, p<0.001), as home therapy (Table 1).

**HOSPITAL ADMISSION SYMPTOMS AND PARAMETERS**. Dyspnea and cough were the most frequent symptoms, being present in 54 (50%) and 51 (47%). Other symptoms reported were diarrhea (13%), nausea (11%), chest pain (9%), syncope (2%), and palpitations (1%). No differences among groups were observed with regards symptoms.

With regard to clinical parameters, median heart rate was 85 beats per minute (IQR 75 – 91), systolic arterial pressure 130 mmHg (IQR 115 – 147), body temperature 37.0 (IQR 36.2 – 37.8), arterial oxygen saturation 96% (IQR 92 – 98), arterial oxygen tension 68 mmHg (IQR 59 – 84), P/F ratio 257 (IQR 159 – 318). Fever was detected in 89 (82%). Non-survivors had a lower arterial oxygen saturation (94 vs 96%, p=0.020) and a lower P/F ratio (231 vs 280, p=0.019), when compared with the survivors (Table 1).

**CLINICAL MANAGEMENT.** Of the 41 patients with abnormal Hs-cTnI on admission, 7 (4 females, median age 86 years old) showed a >20% rise of Hs-cTnI at 3 hours and only one of these patients (91-year-old women) had chest pain and showed ST-segment elevation on electrocardiogram. None of these patients underwent cardiac magnetic resonance, cardiac computed tomography or coronary angiography, because not considered crucial for the patient management or lifesaving. All these patients were managed conservatively, with medical therapy. Five out of 7 patients died.

**Table 1**

|  | **COVID-19** | **Alive** | **Dead** | |  |
| --- | --- | --- | --- | --- | --- |
|  | **n=109** | **n=89** | | **n=20** | **p** |
| **Other Comorbidities** |  |  | |  |  |
| Atrial fibrillation | 21 (19) | 15 (17) | | 6 (30) | 0.211 |
| Chronic pulmonary disease | 17 (16) | 13 (15) | | 4 (20) | 0.548 |
| Chronic liver disease | 7 (6) | 7 (8) | | 0 | 0.345 |
| Solid Malignancy | 16 (15) | 11 (12) | | 5 (25) | 0.167 |
| Leukemia | 1 (0.6) | 0 | | 1 (5) | 0.183 |
| Lymphoma | 1 (0.6) | 1 (1) | | 0 | 1.000 |
| Acquired immunodeficiency disease | 0 | 0 | | 0 | - |
| Connective tissue disease | 6 (6) | 6 (7) | | 0 | 0.590 |
| Dementia | 17 (16) | 11 (12) | | 6 (30) | 0.082 |
| Peripheral atheromasia | 23 (21) | 15 (17) | | 8 (40) | **0.033** |
| **Home therapy** |  |  | |  |  |
| ACEi/ARB | 47 (43) | 38 (43) | | 9 (45) | 1.000 |
| Beta Blockers | 25 (23) | 16 (18) | | 9 (45) | **0.011** |
| Statins | 28 (26) | 22 (25) | | 6 (30) | 0.500 |
| Diuretics | 21 (19) | 11 (12) | | 10 (50) | **<0.001** |
| Cardioaspirin | 25 (23) | 19 (21) | | 6 (30) | 0.309 |
| P2Y12 Inhibitors | 10 (9) | 8 (9) | | 2 (10) | 0.683 |
| Oral anticoagulants | 13 (12) | 9 (10) | | 4 (20) | 0.405 |
| **Admission parameters and symptoms** | |  | |  |  |
| Hear rate, beat per minute | 85 (75 – 91) | 85 (74 – 90) | | 90 (77 – 100) | 0.119 |
| Systolic arterial pressure, mmHg | 130 (115 – 147) | 130 (115 – 144) | | 135 (105 – 154) | 0.695 |
| Body temperature, °C | 37 (36.2 – 37.8) | 37.2 (36.5 – 38.1) | | 36.8 (36 – 37.5) | 0.153 |
| Respiratory rate, breath per minute | 22 (20 – 26) | 22 (20 – 26) | | 24 (20 – 30) | 0.198 |
| Arterial oxygen saturation, % | 96 (92 – 98) | 96 (93 – 98) | | 94 (89 – 97) | **0.020** |
| Arterial oxygen tension, mmHg | 68 (59 – 84) | 70 (61 – 85) | | 64 (56 – 83) | 0.081 |
| P/F ratio | 257 (159 – 318) | 280 (172 – 333) | | 231 (112 – 261) | **0.019** |
| Fever | 89 (82) | 76 (92) | | 13 (77) | 0.089 |
| Dyspnea | 54 (50) | 42 (51) | | 12 (71) | 0.132 |
| Cough | 51 (47) | 43 (52) | | 8 (47) | 0.721 |
| Diarrhea | 14 (13) | 14 (17) | | 0 | 0.119 |
| Nausea | 11 (11) | 10 (12) | | 1 (6) | 0.684 |
| Chest pain | 7 (9) | 6 (10) | | 1 (5) | 1.000 |
| Syncope | 2 (2) | 2 (2) | | 0 | 1.000 |
| Palpitations | 1 (1) | 0 | | 1 (5) | 0.170 |

Categorical variables are presented as number of patients (%). Continuous values are expressed as median with 25% and 75%-iles. Abbreviations: ACEi= ACE inhibitors; ARB= angiotensin receptor blockers;
